# Loss-less Nano-fractionator for High Sensitivity, High Coverage Proteomics * [Fn FN2]

**DOI:** 10.1074/mcp.O116.065136

**Published:** 2017-01-26

**Authors:** Nils A. Kulak, Philipp E. Geyer, Matthias Mann

**Affiliations:** From the ‡Department of Proteomics and Signal Transduction, Max Planck Institute of Biochemistry, Martinsried, Germany;; §PreOmics GmbH, Martinsried, Germany; and; ‖Novo Nordisk Foundation Center for Protein Research, Faculty of Health Sciences, University of Copenhagen, Copenhagen, Denmark

## Abstract

Recent advances in mass spectrometry (MS)-based proteomics now allow very deep coverage of cellular proteomes. To achieve near-comprehensive identification and quantification, the combination of a first HPLC-based peptide fractionation orthogonal to the on-line LC-MS/MS step has proven to be particularly powerful. This first dimension is typically performed with milliliter/min flow and relatively large column inner diameters, which allow efficient pre-fractionation but typically require peptide amounts in the milligram range. Here, we describe a novel approach termed “spider fractionator” in which the post-column flow of a nanobore chromatography system enters an eight-port flow-selector rotor valve. The valve switches the flow into different flow channels at constant time intervals, such as every 90 s. Each flow channel collects the fractions into autosampler vials of the LC-MS/MS system. Employing a freely configurable collection mechanism, samples are concatenated in a loss-less manner into 2–96 fractions, with efficient peak separation. The combination of eight fractions with 100 min gradients yields very deep coverage at reasonable measurement time, and other parameters can be chosen for even more rapid or for extremely deep measurements. We demonstrate excellent sensitivity by decreasing sample amounts from 100 μg into the sub-microgram range, without losses attributable to the spider fractionator and while quantifying close to 10,000 proteins. Finally, we apply the system to the rapid automated and in-depth characterization of 12 different human cell lines to a median depth of 11,472 different proteins, which revealed differences recapitulating their developmental origin and differentiation status. The fractionation technology described here is flexible, easy to use, and facilitates comprehensive proteome characterization with minimal sample requirements.

Mass spectrometry (MS)-based bottom-up proteomic workflows consist of multiple steps, namely sample preparation, on-line liquid chromatography (LC) coupled with MS measurements, followed by computational data analysis, and interpretation. LC-MS/MS technologies have improved drastically from the initial identification of one or a few proteins using manual, complex, and time-consuming protocols to essentially complete proteomic coverage of microorganisms in a rapid and streamlined manner (1–4). These advances are based on multiple breakthroughs in the analytical and computational sides of the proteomic workflow over the last decade and now make MS-based proteomics a powerful player in systems biology (5). However, for complex proteomes, such as human cell lines, organs, and body fluids, very deep characterization still involves great effort, sample amounts, and costs. Therefore, there is a continuing need for more powerful workflows, and here we contribute to these efforts in the important area of peptide pre-separation before the LC-MS/MS analysis.

To yield in-depth proteomes of complex biological samples, two-dimensional separation approaches at the peptide level are attractive because they are more universally applicable than protein level fractionation. First dimension separation techniques range from isoelectric focusing (6–9) and pipette-based approaches such as StageTip fractionation (10–12) to off-line HPLC systems (13–17). High pH reversed-phase fractionation, alternatively termed basic reversed-phase, as a first off-line chromatography separation in combination with the low pH reversed-phase fractionation in the second on-line dimension was first demonstrated more than 10 years ago. In comparison with other methodologies, it benefits from the uniform first dimensional peptide elution profiles achievable with high pH reversed-phase separation and the high peptide separation efficiency in both dimensions (18, 19). Because the two separation dimensions are not completely orthogonal (meaning that peptide retention times are still correlated), direct application of high pH fractionation would lead to non-uniform filling of the gradient in the second dimension. The key advance that solved this problem was the combination of fractions that elute at substantially different times in the first dimension (13). This “concatenation” tends to uniformly fill the gradients, leading to deeper proteomes independent of the nature of the sample, while maintaining throughput (20–22). This two-dimensional separation technique, combining high pH fractionation with concatenation, compares favorably with other approaches and is increasingly being applied by the proteomics community (20, 23–27).

Despite the success of high pH reversed-phase fractionation in the deep characterization of complex proteomic samples, a current limitation is the requirement for rather large amounts of starting material. This is due to the large column diameters, flow rates, and number of fractions that are collected before concatenation to preserve peak separation from the first dimension and to maintain collection volumes that can easily be handled. Therefore, instead of the nanoflow systems typical of on-line separation, much larger columns and flow rates are almost always employed. This in turn requires large sample sizes, and milligram amounts of starting material are typical for high pH reversed-phase fractionation. Unfortunately, this implies high reagent costs, for instance for proteolytic enzymes or for the chemical labeling reagents used in multiplexing. Furthermore, it restricts deep proteomes preliminary to cases where comparatively large protein amounts are available and excludes the investigation of rare cellular subpopulation or laser micro-dissected cells in tumor tissues, for instance. High pH fractionation with higher flow rates and larger sample amounts is also used to investigate post-translational modifications in great depth by the combination of isobaric mass tag labeling of peptides after digestion, followed by high pH fractionation and consecutive enrichments (28). In such cases, large sample amounts and high volumes are necessary because of the subsequent enrichment. However, post-translational modification analysis could benefit from a drastic scale-down in high pH fractionation, if already enriched peptides are fractionated. This would necessitate high sensitivity of the fractionation step and be economically attractive in terms of labeling reagents.

Here, we describe a novel approach that allows efficient sample concatenation without using large volumes. Instead, nanoflow systems are employed, and the intermediate sample collection step is eliminated. We demonstrate the operating principle of our spider fractionator, show that fractionation efficiency remains very high, and establish that the flexibility of the system allows choosing an optimum balance between measurement time and desired depth of proteome coverage. Very low sample amounts can be separated without apparent fractionation-induced sample losses. We demonstrate the sensitivity of the system by the analysis of 12 human cell lines to a depth of about 10,000 proteins with only 1 μg of sample.

## MATERIALS AND METHODS

### 

#### 

##### Cell Culture

HeLa cells were cultured in high glucose DMEM with 10% fetal bovine serum and 1% penicillin/streptomycin (all from Life Technologies, Inc.). Cell lines were cultured in Dulbecco's modified Eagle's medium (Invitrogen) containing 10% dialyzed fetal bovine serum and penicillin/streptomycin. Cells were counted using a countess cell counter (Invitrogen), and aliquots of 10^6^ cells were snap-frozen and stored at −80 °C.

##### Tryptophan Fluorescence Emission Assay for Protein Quantification

Protein concentrations were determined after solubilizing the samples in 8 m urea by tryptophan fluorescence emission at 350 nm using an excitation wavelength of 295 nm. Tryptophan at a concentration of 0.1 μg/μl in 8 m urea was used to establish a standard calibration curve (0–4 μl). From this, we estimated that 0.1 μg/μl tryptophan are equivalent to the emission of 7 μg/μl of human protein extract, assuming that tryptophan on average accounts for 1.3% of human protein amino acid composition.

##### Sample Preparation, Protein Digestion, and in-StageTip Purification

Sample preparation was performed as described previously (3) with the following adaptations. 300 μg of cells were suspended in 50 μl of SDC reduction and alkylation buffer (3). We used 2-chloro-N,N-diethylacetamide as alkylating reagent for the comparison of the 13 cell lines and 2-chloro-acetamide for all other experiments. The cells were kept at 95 °C for 5 min to denature proteins and afterward sonicated to shear DNA and enhance cell disruption with a water bath sonicator (Bioruptor, model UCD-200, Diagenode) for 15 min at the maximum level. The proteolytic enzymes LysC and trypsin were added in a 1:100 ratio (micrograms of enzyme to micrograms of protein), and the solution was incubated for 4 h at 37 °C.

Peptides were acidified by adding 100 μl of ethyl acetate, 1% TFA and extensive mixing for 2 min, and 20 μg were transferred into StageTips containing two 14-gauge SDB-RPS (poly(styrene-divinylbenzene) reversed phase sulfonate) plugs. Afterward, the StageTips were washed with 100 μl of ethyl acetate, 1% TFA to strip SDC and lipids from the digested cells. This was followed by a wash step with 100 μl of ddH_2_O ^1^, 0.2% TFA. The purified peptides were eluted with 60 μl of 80% acetonitrile, 19% ddH_2_O, 1% ammonia in autosampler vials. For all steps, the StageTips were centrifuged at 2,000 × *g* until the solutions were rinsed through completely. The collected material was dried using a SpeedVac centrifuge at 60 °C (Eppendorf, Concentrator Plus). Peptides were suspended in 2% acetonitrile, 0.1% TFA in ddH_2_O and sonicated for 15 min in a water bath sonicator (Branson Ultrasonics, Ultrasonic Cleaner Model 2510). Moreover, 6,600 HeLa cells, the equivalent to 1 μg of starting material (29), were separately digested using the in-StageTip protocol (3) with the above mentioned adaptations.

##### Pre-fractionation

We constructed a software-controlled, fully automated, rotor-valve-based fraction collector system coupled on line to a nanoflow HPLC (EASY-nLC 1000 system, Thermo Fisher Scientific), and we used this for all high pH reversed-phase pre-fractionations. The fraction collector system was named Spider Fractionator and is under commercial development by PreOmics GmbH, Martinsried, Germany. We provide a list of components used in constructing the fractionator (supplemental Table 1). For the work reported here, we standardized on a first dimension column of 250 μm inner diameter and a length of 30 cm, which was packed with 1.9 μm C18 particles (ReproSil-Pur C18-AQ 1.9 μm resin by Dr. Maisch GmbH) and has an estimated loading capacity of at least 100 μg. The column is available from PreOmics GmbH (Article No. P.O. 00007). All columns (first dimension and on-line dimension) were passivated by a single run of BSA to saturate irreversible binding sites. For separation into eight pooled fractions, we loaded 20 μg (or other amounts where indicated) of purified and digested peptides onto a reversed-phase C_18_ column. A gradient was generated by using a dual buffer system (buffers A and B) also from PreOmics GmbH (Article No. P.O. 00009). Peptides were eluted from 3% B to 30% B in 45 min followed by a linear increase to 60% B in 17 min. This gradient was followed by a further linear increase to 95% B in 5 min and a 3 min wash at 95% B, followed by a 10 min decrease to 3% B. The last segments ensure that the output lines (volume about 800 nl) are emptied, and none of the remaining peptides are lost. The flow rate was kept at a constant 2 μl/min. The 96-well plate was moved by a stepper motor-driven linear actuator. Software was implemented on a Raspberry microcontroller.

We separated peptides into 4, 8, 16, and 24 fractions using rotor valve shifts of 90 s. Fractions were collected into 0.2-ml thin-walled 8-tube strips (Thermo Fisher Scientific). We loaded 20 μg of starting material for 4 and 8 fractions, 40 μg for 16 fractions, and 60 μg for 24 fractions.

The concatenation scheme of table I was used for pooling. For a more detailed version of the fractionation schemes see supplemental Fig. 1.

**Table I:** Concatenation scheme

No. pooled fractions	4	8	16	24
Peptide amount (μg)	20	20	40	60
No. of non-pooled fractions	54	54	54	54
Pooling scheme	1;5;9;13;17;21;25;29;33;37;41;45;49;53	1;9;17;25;33;41;49	1;17;33;49	1;25;49
	2;6;10;14;18;22;26;30;34;38;42;46;50;54; etc.	2;10;18;26;34;42;50; etc.	2;18;34;50; etc.	2;26;50; etc.

The pooled fractions were dried using a SpeedVac centrifuge at 60 °C (Eppendorf, Concentrator Plus). Peptides were suspended in 2% acetonitrile, 0.1% TFA in ddH_2_O and sonicated for 15 min in a water bath sonicator (Branson Ultrasonics, Ultrasonic Cleaner Model 2510). A total of 2 μg of each concatenated fraction was loaded and measured by LC-MS/MS as described below.

##### Comparison of the Spider Fractionator to Other High pH Fractionation Systems

We used the same buffers, gradients, and pooling scheme as for the spider fractionator system in comparison with a higher flow system and to a recently introduced spin column system. For all three systems, the same HeLa digest was used to fractionate 1 or 20 μg of peptides. The higher flow system consisted of an XBridge peptide BEH C18 column (2.5 μm particle size, 2.1 × 250 mm, Waters) with a Shimadzu HPLC system at a 60 °C run at a flow rate of 150 μl/min. The fractions were manually pooled. For the 1 μg sample, all fractions were re-pooled into a single vial to determine sample loss. For the 20 μg sample, we manually concatenated samples according to the same scheme as automatically done by the spider fractionator. On the spin system (high pH reversed-phase peptide fractionation kit, Pierce catalog number 84868), separation was done according to the manufacturer's instructions resulting in eight fractions but no concatenation.

##### Ultra-high Pressure Liquid Chromatography and Mass Spectrometry

Samples were measured using LC-MS instrumentation consisting of an EASY-nLC 1000 ultra-high pressure system (Thermo Fisher Scientific) coupled via a nanoelectrospray ion source (Thermo Fisher Scientific) to a hybrid quadrupole Orbitrap mass spectrometer (Q Exactive HF Orbitrap from Thermo Fisher Scientific) (30, 31). Purified peptides were separated on 40 cm HPLC columns (75 μm inner diameter; in-house packed into the tip) at 60 °C with ReproSil-Pur C18-AQ 1.9 μm resin by Dr. Maisch GmbH).

For all measurements, peptides were loaded in buffer A (0.1% formic acid, 5% DMSO (32)) and eluted with a linear 55 min gradient of 2–20% of buffer B (0.1% formic acid, 5% DMSO, 80% acetonitrile), followed by an increase to 40% buffer B within 40 min and afterward within 2 min to 98% buffer B and a 2 min wash at 98% buffer B. The flow rate was kept at 350 nl/min.

Column temperature was kept at 60 °C by an in-house-developed oven containing a Peltier element, and parameters were monitored in real time by the SprayQC software (33).

MS data was acquired with the Thermo Xcalibur software version 3.0.63, a topN method where *N* could be up to 100. This method in principle allows a very large number of precursor peaks to be picked for fragmentation but is in practice limited by the number of precursors with sufficient ion intensity. In the entire data set, *N* was 15 on average. Target values for the full scan MS spectra were 3 × 10^6^ charges in the 300–1,650 *m*/*z* range with a maximum injection time of 15 ms. Transient times corresponding to a resolution of 60,000 at *m*/*z* 200 were chosen. A 1.5 *m*/*z* isolation window and a fixed first mass of 100 *m*/*z* were used for MS/MS scans. Fragmentation of precursor ions was performed by higher energy C-trap dissociation (34) with a normalized collision energy of 27 eV. MS/MS scans were performed at a resolution of 15,000 at *m*/*z* 200 with an ion target value of 5 × 10^4^ and a maximum injection time of 25 ms. Dynamic exclusion was set to 30 s to avoid repeated sequencing of identical peptides.

##### Data Analysis

MS raw data files were analyzed by MaxQuant software version 1.5.3.31 (35), and peptide lists were searched by the Andromeda search engine (36) against the human Uniprot FASTA database to which common contaminant proteins had been added (86,746 entries) with cysteine diethylcarbamidomethylation as a fixed modification for the comparison of the 13 cell lines and cysteine carbamidomethylation as a fixed modification for all other experiments. N-terminal acetylation and methionine oxidations were used as variable modifications in all experiments. The false discovery rate was set to 0.01 for both proteins and peptides with a minimum length of 7 amino acids and was determined by searching a reverse database. Enzyme specificity was set to trypsin and a maximum of two missed cleavages were allowed in the database search. Peptide identification were performed with an allowed initial precursor mass deviation up to 7 ppm and an allowed fragment mass deviation of 20 ppm. The MaxQuant feature “match between runs” was enabled within the dataset of the pooled eight fractions and the single shot samples for all cell line samples. Proteins matching the reversed database were filtered out. Label-free protein quantification was done with a minimum ratio count of 1 (37). All bioinformatics analyses were performed within the Perseus software of the MaxQuant computational platform (35, 37).

## RESULTS

### 

#### 

##### Spider Fractionator

**Fig. 1.**
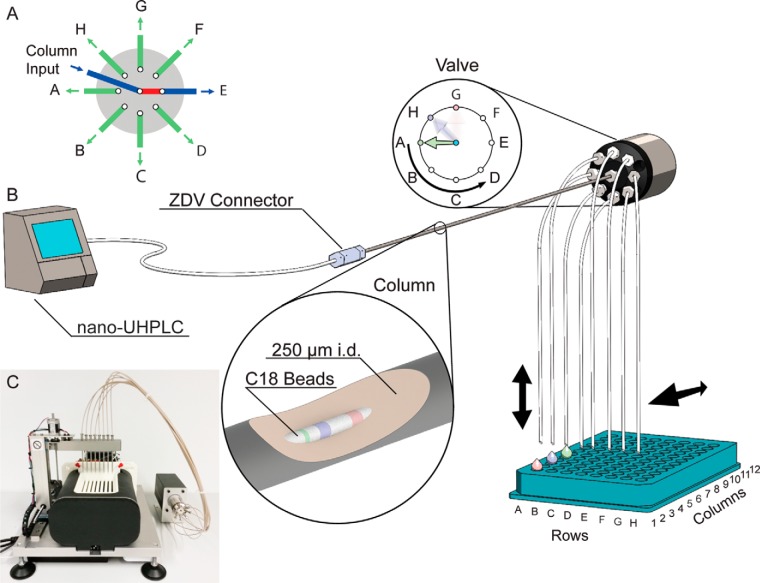
**Spider fractionation principle and practical implementation.** *A*, switch mechanism of the rotor valve, illustrating how the flow from the first dimension separation is divided to eight output lines. *B*, schematic of the implementation of the spider fractionator. The first dimension separation is realized as a 250 μm inner diameter column, connected upstream through a zero dead volume connector to a nano-HPLC pump (an ultra high pressure unit is depicted but not required). The zoom-in is a cut-away symbolizing different peptide bands being separated in the column by different colors. Downstream, the column is connected to the rotor valve from *A*. The output lines feed into tubes that are filled in turn, according to the concatenation scheme. The spider-like appearance of the output lines give the name to the device. The *arrows* indicate that the output lines can be moved to a new set of tubes for a new separation process. After separation, the tubes are inserted into the autosampler of an UHPLC for LC-MS/MS analysis of the fractions. *C*, photo of the prototype spider fractionator used in this work.

The principle of the fractionator is depicted in Fig. 1. The post-column flow from the first dimension separation enters the input port of an eight-port flow-selector rotor valve. At pre-determined time intervals, the valve switches to a new output port. Each of the outputs is connected via a narrow bore capillary to different output lines in a sample collection device, distributing the sample flow into consecutive tubes for the pooled fractions. Once one cycle has been completed, the valves switches back to the first output port and the next fluid volume is added to the already collected first fraction. In this way, the device automatically concatenates and pools the samples, without requiring different collection tubes or the combination of separately collected effluent volumes. Therefore, the volumes are not constrained to a minimum size, which would otherwise be necessary to handle them in separate tubes. We routinely employ a 250 μm inner diameter column in the first dimension at 2 μl/min and switch the valve every 90 s, thus concatenation volumes are only 3 μl. The system is fully programmable, allowing collection not only into multiples of the eight output channels (A–H) but also into as few as two or as many fractions as there are collection tubes in the device (96 in our setup). Furthermore, an arbitrary number of samples can be fractionated, and the rotor valve shifts can be defined by the user. For example, 12 samples could be scheduled for concatenation into eight fractions each in a total of 24 h using 80 min gradients.

When operating in a high pH reversed-phase mode, we use the first column with a buffer at pH 10, which is devoid of non-volatile constituents. The column inner diameter and consequently the flow rate are chosen such that the desired peptide amounts are present in the sample tubes after concatenation. For instance, using second dimension columns with a loading capacity of 2 μg, which is typical of the 75 μm inner diameter columns used in many proteomics laboratories, would call for a sample amount of at least 16 μg to be loaded on the first dimension column. Therefore, the first column should have a capacity of at least the second column multiplied by the number of desired fractions. In principle, the entire system can be scaled up or down as required. Within the constraints mentioned above, different size columns and separation principles can be combined as long as they are at least partially orthogonal. For the work reported here, we standardized on a first dimension column of 250 μm inner diameter and a length of 30 cm, which is packed with 1.9 μm C_18_ particles and has an estimated loading capacity of at least 100 μg (see “Materials and Methods”).

For the subsequent on-line LC separation, no alterations compared with standard laboratory procedures are necessary. In the work reported here, the columns were 40 cm long, 75 μm inner diameter, and packed with 1.9 μm C_18_ particles. The 0.1% formic acid in our buffers ensured low pH compared with the first dimension.

The overall fractionation systems was realized by coupling an EasyLC nanobore HPLC to the first dimension column (see “Materials and Methods”). Note that back-pressure was only 250 bar. Because no high pressure capability is required, a wide range of nanoflow pumps used in proteomics would therefore be suitable. The fractionator principle itself is embodied in an apparatus containing a column oven to maintain 60 °C, the flow-selector valve for fractionation, the required column, two-dimensional axes for automated multi-collection plate position selection, a cooling unit to retain fractions at about 6 °C, a microprocessor control unit for automated contact closure and HPLC interaction, and a driver software to control, log, and monitor all the parameters (Fig. 1*C*). The control unit maintains communications to the upfront HPLC system, to the rotor valve, and to the downstream fraction collection system. The collection system is designed to be fully flexible. Peptides eluting from the column are separated into packages defined by a time interval by rotor valve shifts. The shift in valve position directs each package into one of the eight output lines. Each of these are placed into one of the eight “rows” (A–H) of a 96-well layout. Output line A elutes into row A, line B into row B, and so forth. Eight shifts of the rotor valve will deposit peptides from the column into each tube of the first column, and the next shift will enter the next output line and therefore again fill the first row A. In this way, for eight or less fractions, the output line holder stays at column 1 of the 12 possible positions of a 96-well plate. In case separation into more than eight fractions is desired, the output line holder will move from column 1 to column 2 after eight packages (H1 is followed by A2, supplemental Fig. 1). For 16 fractions, the output holder will move back to column 1 after eight rotations (H2 is followed by A1). Likewise, 24, 32, 40, 48, 56, and 96 fractions can be realized. Fractionation into less than eight fractions or any other desired number between 1 and 96 is also possible as the rotation valve can be programmed to direct packages to arbitrary output lines. For instance, in the case of four fractions, the rotation valve switches directly from D1 to A1. The collection tubes are maintained cooled and can be placed in a SpeedVac and subsequently into the auto sampler of the on-line LC-MS/MS system.

##### Separation Performance

**Fig. 2.**
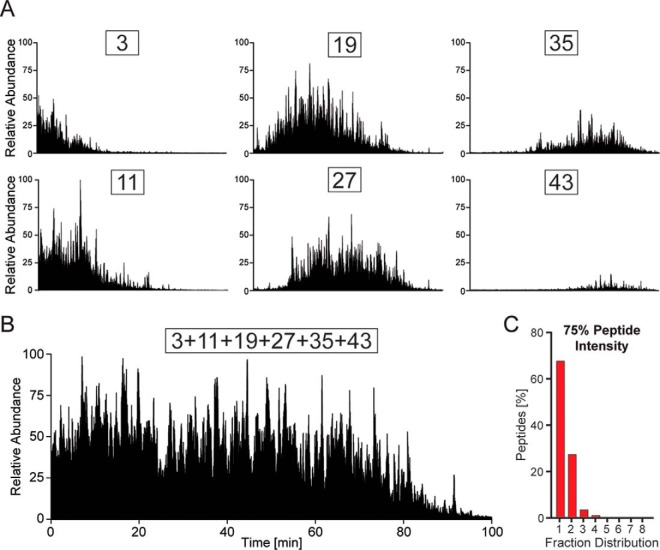
**Comparison of pooled and non-pooled peptide mixtures and separation efficiency.** *A*, total ion current of separately collected, 90-s elution cuts from the 1st dimension column. *B*, total ion current of automatically pooled fractions corresponding to the ones in *A. C, histogram* of peptides containing at least 75% of their total mass over all fractions in the indicated number of fractions.

With the column connected to the Spider fractionator, we first collected each of 54 fractions (90 s duration) in their own tubes. Starting from fraction three, we chose every 8th fraction and analyzed these fractions separately in 100 min gradients on the 40 cm analytical column. The 90 s elution windows from the first dimension eluted roughly in the same region as expected if they had been separated on a low pH analytical column except that their elution range was expanded considerably due to the different pH values (Fig. 2*A*). However, generally the bulk of the peptides was still concentrated within only about 20–50% of the total gradient.

Next, we specified an eight fraction concatenation scheme, meaning that the rotor valve combined the 54 fractions into eight equally filled gradients. Fractions 3, 11, 19, 27, 35, 43, and 51, which were measured separately above, were automatically combined by the rotor valve. Analyzing this concatenated fraction on the 100 min gradient of the analytical column resulted in a peptide elution profile that was filled over the entire range, resembling the super position of the separately measured fractions (Fig. 2*B*). Repeating this experiment in triplicate yielded essentially identical elution profiles, demonstrating reproducibility of the spider fractionator (supplemental Fig. 2).

A desirable feature of a pre-fractionation apparatus is that it concentrates each individual peptide into as few fractions as possible. Note that in a two-dimensional separation scheme there will always be peptides that will be collected into different tubes because the fractionation will occur during peak elution for a certain percentage of them (“peak cutting”). Furthermore, peptides may or may not be sequenced and identified in different fractions, depending on the complexity of the sample and the sequencing speed and sensitivity of the mass spectrometer. Therefore, it is necessary to quantify peptide abundance across all fractions. To evaluate the separation efficiency of the fractionator and pooling scheme, we therefore employed the “match between runs” option in the MaxQuant software (11, 38), which allowed the transfer of identifications between the fractions. Note that the label-free algorithms in MaxQuant in any case normalize the contributions of the fractions and add the contributions for peptides found in more than one (37).

For the majority of peptides (68%), their total intensities were concentrated in one fraction to more than 75% (Fig. 2*C* and supplemental Fig. 3). This is roughly in line with a model in which the peptide distribution in the analytical dimension is largely a function of the cutting of peaks in the first dimension. (In our case, a peak width of 15 s in the first dimension and a 90 s collection window would result in about 15/90 = 16.6% of “cut peaks”).

##### Evaluating the Optimal Number of Fractions

For any sample, the spider fractionator allows choosing the desired number of fractions. A large number of fractions will increase proteome coverage in two ways. First, at any chosen gradient length, the time available for sequencing peptides will increase with the number of fractions. In complex samples, this will lead to more identified peptides and proteins. Second, as there is a maximal loading capacity of the analytic column, a larger number of total fractions increases the total material that can be used in a proteomic analysis and therefore its sensitivity. Conversely, many fractions imply long measurement times per sample and may be less beneficial if sample size is limited. In practice, a good compromise, maximizing the effort/gain balance, needs to be found according to the parameters and the goals of the experiment at hand.

To investigate this, we employed HeLa digest as a typical complex proteome and determined the number of identified peptides and proteins as a function of fraction number. We separated peptides into 4, 8, 16, and 24 fractions. We loaded 20 μg of starting material for 4 and 8 fractions, 40 for 16 fractions, and 60 for 24 fractions, so as not to be sample limited for the individual LC MS/MS runs, in which an estimated 2 μg were injected in each case. As expected, separation into 24 fractions, followed by 48 h of total MS measurement time, yielded the largest number of different peptides and proteins groups. In total, 128,966 sequence unique peptides and 10,769 different protein groups were identified by MaxQuant in the HeLa cells with 1% false discovery rate at the protein and peptide levels. Match between runs to all files acquired in this project increased these numbers to 159,024 sequence unique peptides and 11,897 protein groups (supplemental Table 2).

**Fig. 3.**
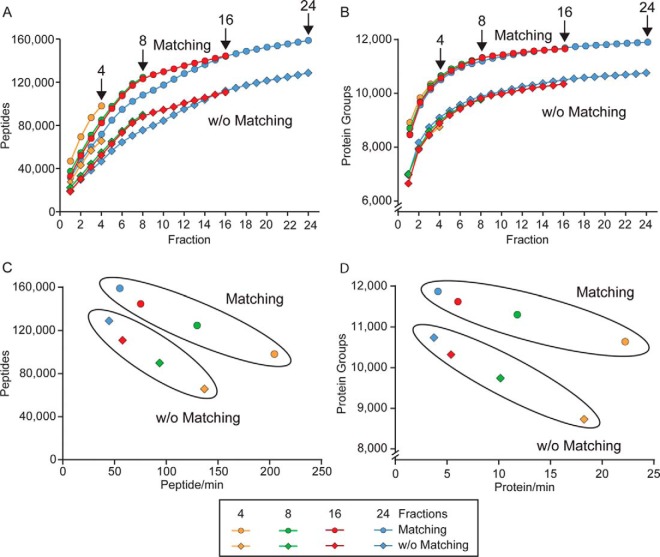
**Effect of different numbers of pooled fractionations on proteome coverage.** *A*, cumulative number of sequence unique peptides as a function of fraction number for a 4, 8, 16, and 24 fractionation scheme. The *upper curves* (*circles*) are obtained with match between runs enabled in MaxQuant and the *lower curves* (*diamonds*) without match between runs. The last fraction of the experiment is labeled in each case. *B*, same as *A* but for protein numbers. *C*, number of peptides identified per min in 100 min gradient runs as a function of total number of peptides identified. Values enclosed in the *upper ellipse* are those employing match between runs and in the *lower ellipse* without match between runs. High values on the *x* and on the *y* axis are desirable (large number of identifications per min as well as high number of identified peptides). *D*, same as *C* but for protein numbers.

Strikingly, using 16 fractions (32 h measuring time) and 8 fractions (16 h) still resulted in 98 and 95% of those protein identifications, respectively. Even the four fraction experiments identified 90% of the proteins in 8 h, corresponding to only 1/6th of the measuring time of the 24 fractions. However, although the loss of protein identifications was very moderate with decreasing fraction number, this was not as pronounced at the peptide level, where only 91, 78, and 62% of peptides were still found (supplemental Table 2). This observation is explained by the fact that increasing depth of measurement will result in a saturating number of identified proteins, whereas the number of peptides and the sequence coverage of the proteins still increase. Accordingly, Fig. 3*A* shows a rapid rise of identified peptides when accumulating the results of subsequent fractions within one experiment. The first fractions of each experiment add newly identified peptides at an almost linear rate. Here, the four-fraction experiment has a clear advantage as it identifies 28,000 peptides (47,000 with matching) in the first fraction, whereas the first fraction of the 24-fraction experiment only results in 19,000 peptides (32,000 with matching). This reflects the fact that in the four-fraction experiment each of the fractions contains a quarter of total peptides, whereas the 24-fraction experiment leads a smaller number of indefinable peptides despite the higher amount per peptide. The total number of peptides identified in the four-fraction experiment is already matched between 6 and 7 fractions for the 24-fraction experiment, which goes on to yield almost twice the total number of peptides. At the protein level, the identification numbers are essentially only a function of the number of fractions, and that is to say the cumulative number of proteins per fraction are almost identical. The saturation of the curve has largely occurred by fraction 8, especially when using match between runs (Fig. 3*B*).

For the decision of how many fractions the experimenter should choose, the total number of proteins or peptides as well as the effort/gain balance need to be considered, as already mentioned above. For this purpose, we plotted the total number of proteins and peptides against the peptide- or protein-to-time ratio (Fig. 3, *C* and *D*). Again, it appears that eight fractions result in an optimum regarding both factors.

##### Comparison of the Spider Fractionator to Other High pH Fractionation Systems

The spider fractionator setup was compared with a high flow system (150 μl/min) coupled to a 2.1 mm × 250 mm C18 column and to a recently released spin column-based high pH reversed-phase peptide fractionation kit (see under “Materials and Methods”).

To analyze potential sample losses, we fractionated 1 μg of peptides from the same HeLa digest on all three systems, combined the total eluted volume, and compared the median peptide intensity to a measurement of 1 μg of the same unfractionated peptides. For such low sample amounts, the high flow and the spin column system resulted in much less recovered peptides than the spider fractionator (recoveries were 15, 24, and 81% of the unfractionated sample) (supplemental Fig. 4*A*). Moreover, we fractionated 20 μg of the same HeLa digest with all three systems and compared the median peptide intensities, numbers of identified peptides, and protein groups. The spin column setup allowed only fractionation into eight samples without any concatenation, and for the high flow system the samples were concatenated manually. The spider setup resulted in the highest median peptide intensity, identified peptides, and protein groups, followed by the high flow and the spin column system (supplemental Fig. 4, *B–F*).

The experiments for 1 and 20 μg fractionation amounts point in the direction that the spider fractionator had by far the lowest sample loss. Major sample losses could have occurred due to the interaction surfaces in the high flow and the spin column systems.

Moreover, the fully automated concatenation of the spider fractionator saved a lot of hands-on time compared with the two other systems. The major bottlenecks of the high flow system were losses by handling the high volumes and several pipetting and concatenation steps as well as the very long SpeedVac times of up to 6 h for the 12 ml of fractionated volume.

##### Spider Fractionator Allows Loss Less Fractionation

Because of sample losses associated with high flow rate HPLC systems, fractionation is generally only employed when large sample amounts are available. However, due to its operating principle, the spider fractionator should not have these limitations. To investigate this potential advantage in detail, we fractionated different amounts of digested HeLa peptides (0.5, 1, 2, 5, 10, 20, 50, and 100 μg) into eight pooled fractions each. To minimize potential issues associated with carry-over, we measured the lowest amounts first and on a new column.

**Fig. 4.**
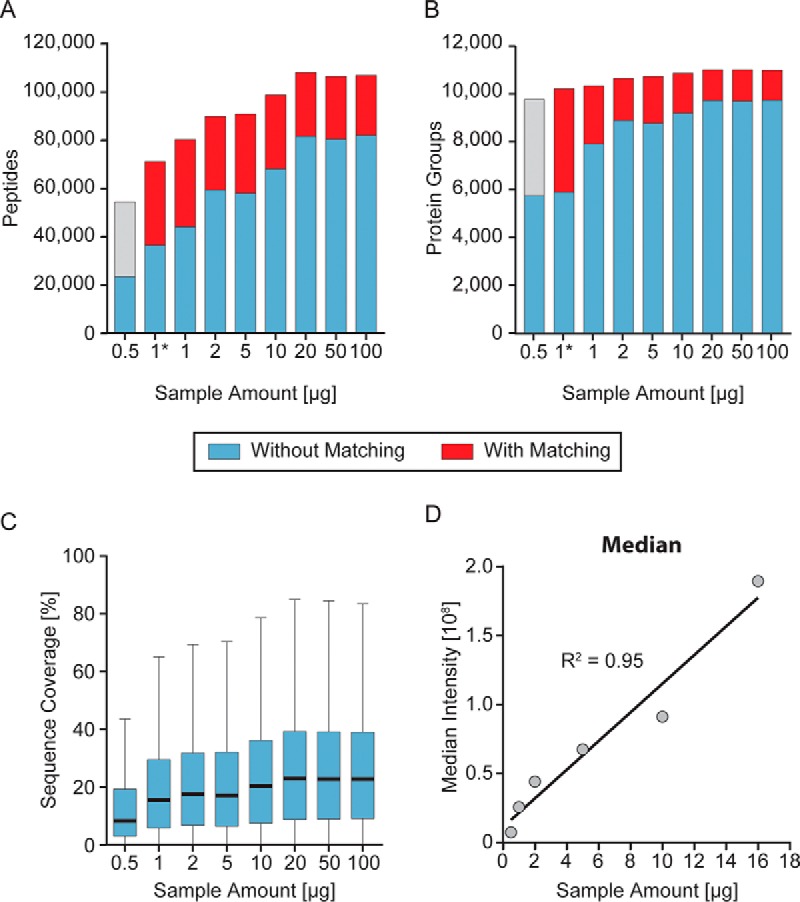
**Dependence of proteome coverage on sample amount.** *A*, fractionation of a total of 0. 5, 1, 2, 5, 10, 20, 50, and 100 μg of HeLa peptides resulted in the indicated number of identified peptides. For the sample amount 1*, we started with 6,600 HeLa cells, which is equal to 1 μg of starting material, for an in-StageTip digestion with subsequent peptide cleanup and fractionation. *Blue* represents peptides identified by MS/MS and *red* those identified by match between runs in MaxQuant. The *gray bar* indicates that the false discovery rate for match between runs was not validated at this very low sample amount. In the case of 20, 50, and 100 μg of starting material, the volume corresponding to 2 μg of peptide material was injected to avoid overloading the analytical column. *B*, same as *A* for the number of identified proteins. *C*, sequence coverage as a function of starting peptide material displayed as Tukey plots. The *bold black lines* represent the median of all proteins. The *blue box* marks the upper and lower quartile of the sequence coverage and the whiskers the 1.5-fold interquartile range. *D*, median intensity determined as label-free intensity values by MaxQuant for all proteins that were quantified in the dilution series are plotted as a function of initial peptide sample amount. Each value is the median of all protein quantifications.

First, we analyzed the behavior at the higher sample amounts. For the three highest sample loadings and assuming an equal distribution of peptides in all fractions, more than 2 μg were available per LC MS/MS run, but only this amount was injected. This yielded the same number of identified peptides and proteins (around 11,000 proteins and nearly 110,000 peptides), demonstrating that the spider fractionator equipped with the 250 μm inner diameter column can handle these amounts of sample or more (Fig. 4, *A* and *B*). The average sequence coverage of the proteome was 26% for fractionation of more 10 μg, decreasing gradually to 20% for 1 μg (Fig. 4*C*).

As expected from the smaller amount of peptide material injected into the analytical column, the total number of peptides identified decreased from 10 to 1 μg of starting material (maximum of 1.25 to 0.125 μg per injection). However, the loss of identification was much less than linear, decreasing to about half with 10-fold lower peptide amount. Remarkably, there was very little loss of protein identifications in the same range. In particular, when using matching, the 1 μg total loading still resulted in more than 10,000 different protein groups (7,800 without matching). Loading less than 1 μg did result in a considerable reduction of proteins and peptides. However, 5,724 proteins were still identified by MS/MS from 23,765 peptides even in this case. Note that this may still not reflect a limitation of the fractionator but instead simply be due to the nanogram amounts of peptide loaded onto the analytical column.

To further investigate the apparent absence of sample losses of the spider fractionator, we next plotted the median intensities of all individual proteins against the amount of injected material up to the 20 μg value (Fig. 4*D*). This resulted in a linear relationship down to the lowest amounts tested, demonstrating that any potential sample losses incurred by the spider fractionator, if they occur at all, are so small that they are not detectable in the setup used here.

To show the applicability of our workflow for a limited amount of starting material, we prepared peptides directly from 6,600 HeLa cells (about 1 μg (29)) by using the in-StageTip protocol (3). The complete material of digested and purified peptides was fractionated using the spider fractionator resulting in 5,869 protein groups and 37,000 peptides without and 10,165 protein groups and 72,110 peptides with matching (Fig. 4, *A* and *B*).

##### In-depth Measurement of Human Cell Lines

Having established optimal fractionation parameters and sample requirements, we next employed the spider fractionator for the in-depth measurement of 12 different human cell line proteomes. Specifically, we used the 8-fraction, 100 min gradient scheme, resulting in a total measuring time of 16 h, including column loading and equilibration and the 20 μg loading, which was the minimum amount that saturates the number of identifiable peptides. Thus, the entire experiment consumed only 8 days of measurement time and much less than a small cell culture dish for each cell line (corresponding to about 100,000 HeLa cells).

**Fig. 5.**
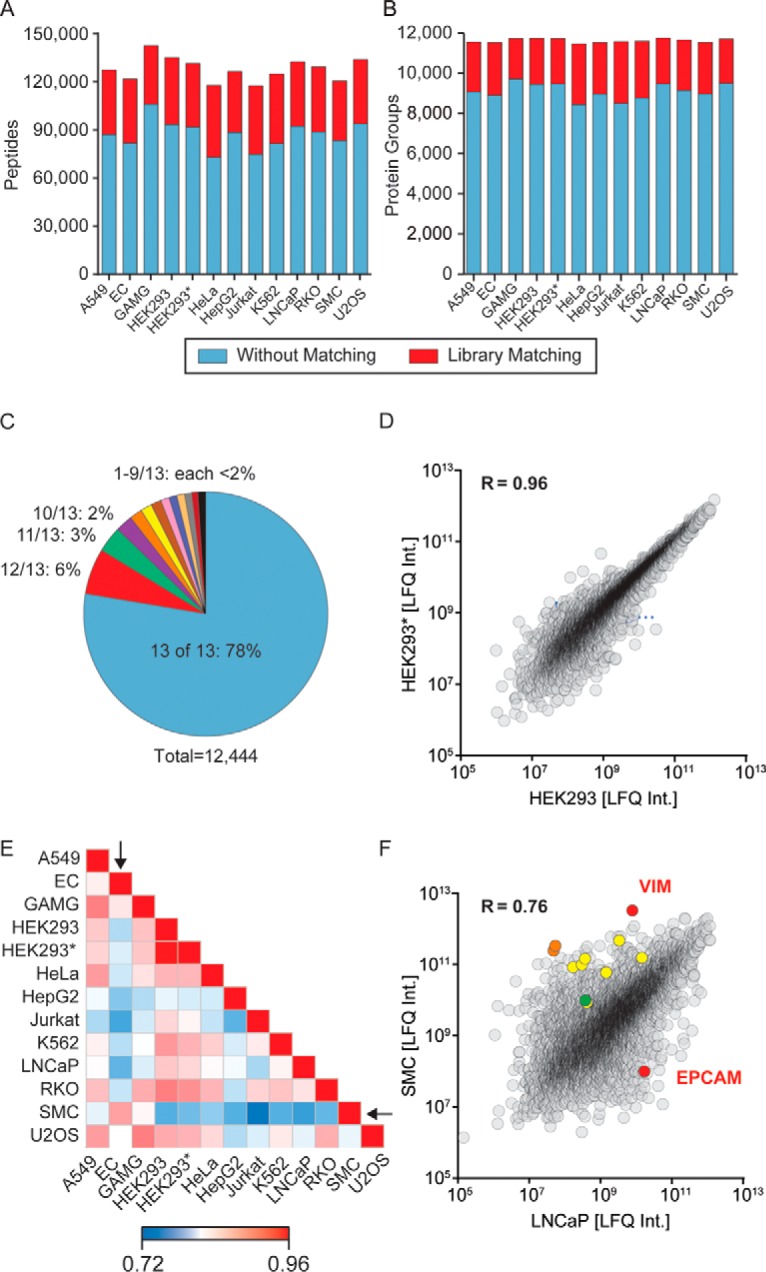
**Rapid and sensitive sequencing of 13 human cell line proteomes.** *A*, number of sequences of unique peptides identified for the different cell lines indicated on the *x* axis (see supplemental Table 2 for cell line abbreviations). *Blue* indicates the proportion identified by MS/MS and *red* the additional peptides identified by match between runs in MaxQuant. *B*, same as *A* for identified protein numbers. *C, pie chart* of the proportion of proteins identified in the indicated number of cell lines. A total of 89% of the proteins identified are also identified in at least 10 of the 13 cell lines. *D*, scatter plot of the label-free intensity (*LFQ*) assigned by MaxQuant to the same protein in two different instances of the same HEK293 cell line (termed HEK293 on the *x* axis and HEK293* on the *y* axis, respectively). *E*, heat map of the rank order correlation of the 13 different proteomes. The SMC and EC cell lines are outliers with respect to their correlations to the others and are indicated by *arrows. F*, scatter plot of the proteins quantified in both the LNCaP (epithelial origin) and the SMC cell line (mesenchymal origin). The known epithelial marker epithelial cell adhesion molecule is much more highly expressed in LNCaP, whereas the known mesenchymal marker vimentin is extremely highly expressed in SMC. Vimentin together with LARP6 (colored in *green*) stabilizes type I collagen mRNA for CO1A1 and CO1A2 (colored in *orange*). Several other collagens (COL12A1, -3A1, -5A1, -6A1, -6A2, -6A3, and -7A1 colored in *yellow*) follow the same pattern.

Combined with the one replicate HEK293 cell line (see below), the experiment yielded a total of 199,882 sequence unique tryptic peptides corresponding to 12,444 different protein groups (supplemental Table 3). The median sequence coverage of these protein groups for this cell line dataset was a remarkable 41.3%. In the 13 cell line experiment the median number of identified peptides was 87,769, and this number ranged from 72,471 in the HeLa sample to 105,487 in the GAMG cell line. Applying the match between run algorithm boosted median peptide identifications by a further 47% (Fig. 5*A*).

The median number of proteins identified in each of the singlet experiments was 11,472, and this was very consistent between cell lines (minimum 11,340 in HeLa and maximum 11,634 in GAMG). These are among the deepest proteome results reported for cell lines so far, which is particularly remarkable given the small sample consumption and measurement time. The proteome of the 13 cell lines (12,444 protein groups; 199,882 peptides) mapped to 11,442 protein-coding genes, which made up more than 57% of the 20,154 entries listened in SwissProt at the time of writing.

The large majority of total identified proteins (78%) was also identified in each singlet experiment and almost 90% of them in at least 10 of the 13 experiments (Fig. 5*C*). This implies that the proteomes of these different cell lines are quite similar in terms of expressed and identifiable proteins. It further implies that our data set, acquired with a data-dependent acquisition strategy, is substantially complete and can only have a very small percentage of missing values, despite the use of data driven shotgun proteomics.

Cell lines, including the ones used here, have often been in culture for years or decades and even those that are nominally the same can develop differences over time. Here we had obtained the human embryonic kidney cell line HEK293 from two institutes and treated them as separate entities for the purpose of comparison with different cell lines. Nearly the same number of proteins as well as peptides was identified after fractionation in both cases, and the overlap of each of the fractionated, matched datasets to all identified proteins was 97.1%, with the unique proteins at the lower levels of expression in proteome. The Pearson coefficient for the abundance rank order of the proteomes calculated from the scatter plot in Fig. 5*D* was 0.96, implying that they were very similar at the quantitative level as well. Thus, a simple fractionation experiment establishes that in this case the same cell lines with different origins are very similar at the proteome level.

We calculated the Pearson correlation between all combinations of the 13 cell lines and plotted the result as a heat map (Fig. 5*E*). As expected, the two instances of the HEK293 cells had the highest correlation, whereas the median correlation was 0.83. Against this very high median correlation, a few cell types show a considerably divergent behavior. One of these is EC, an embryonic carcinoma cell line, whose proteome had a correlation to other cell lines down to 0.77. This observation can be readily explained by the fact that EC is the sole undifferentiated cell line in our set. Interestingly, the only cell line to which EC has a high correlation is SMC, another outlier cell line. The proteome of SMC likewise showed a lower overall correlation to the other cell lines (down to 0.73), and in this case the biological explanation is that muscle is developmentally derived from the mesenchyme, whereas the other cell lines are primarily of epithelial origin. Finally, HepG2 likewise correlates less well than an average cell type, presumably reflecting the specialized organismal role of this model of liver function.

To illustrate how readily acquired deep proteomes can shed light on cellular function, we quantitatively compared SMC against a cell line whose proteome had typical correlation values to the other cell lines. For this, we chose LNCaP, a widely used cell model of prostate cancer. The correaltion between SMC and LNCaP was comparatively poor (R=0.76), and the scatter plot reveals a large number of proteins that were expressed at drastically different levels (Fig. 5*F*). Among these, we found the epithelial cell adhesion molecule, which is the classical positive marker used in immunohistochemistry to stain cells of an epithelial origin, to be strongly increased in LNCaP. Conversely, the classical mesenchymal marker vimentin was strongly expressed in SMC. It is known that vimentin, together with LARP6, stabilizes type I collagen mRNAs, which in turn leads to up-regulation of the collagens CO1A1 and CO1A2 (39). Our data show that several other collagen isoforms are also strongly expressed in this mesenchymal cell line, suggesting that they may be up-regulated by similar mechanisms (Fig. 5*F*).

## DISCUSSION

In the quest for very deep and large scale proteome characterization, pre-fractionation of peptides occupies a pivotal role. We build upon the success of high pH pre-separation as a first dimension coupled to concatenated fractionation sample pooling for the second dimension of analysis. Samples have been separately collected and then combined in these approaches, whereas in the spider fractionator introduced here, concatenation is implemented by a rotating valve. This valve automatically directs sections of the eluent of the first column into a number of tubes corresponding to the number of desired fractions to be analyzed. In this way, any number of pooled fractions with any concatenation volume can in principle be realized. First dimension column diameters and flow rates are much smaller than those typically used in high pH-based proteomics workflows, and the absence of intermediate collection points means that there are no obvious points of sample loss. We implemented the spider fractionator as an assembly of the first dimension column and its accessories, an automated valve, temperature controls, and an automatic fraction collection system for unattended multi-sample fractionation. The device is now routinely used in our laboratory for any project involving pre-fractionation and has proven robust in dozens of projects already.

Here, we characterized the spider fractionator in different dimensions of performance. Comparison of individually combined and pooled samples gave very similar results at high sample amounts, demonstrating the automated pooling scheme correctly implements the concatenated high pH strategy. We obtained quantitative intensity profiles over the pooled fractions for tens of thousands of peptides, which showed that the bulk of each individual peptide mass is localized to a single fraction. The fractionator can be operated in a parameter space defined by the number of fractions and the width of the volume that is concatenated. Likewise, the diameter, flow rate, and stationary phase of its column can be chosen to fit the desired objectives, within at least the range of up to 100 μg, above which a standard high pH setup may be just as effective. Using the same C_18_ material as in our standard LC MS/MS setup, we investigated the influence of the number of fractions on the depth of proteome coverage. Four fractions already led to a very good proteome coverage, and adding additional fractions up to 24 fractions resulted in asymptotic gain at the protein level, while peptide coverage still improved. Considering the tradeoffs in measuring time and available sample quantity in terms of proteins identified per min, we conclude that an eight-fraction scheme is a good compromise in many situations.

Using these parameters, we then demonstrated that the spider fractionator enables extraordinary profiling sensitivity and depth in high pH fractionation experiments. As little as 1 μg of peptide sample, when fractionated, enabled the identification of more than 10,000 proteins. Analysis of protein signal as a function of increased loading of the first dimension column demonstrated that the device has little if any detectable sample loss. We then applied the spider fractionator to the rapid analysis of small amounts of cell line material, a typical challenge for proteomics. In only 16 h we reached a proteome coverage of a median of 11,472 different protein groups (a total of 12,444 different protein goups for all cell lines). In the past, our group employed much longer measurement times and larger sample amounts and still only reached smaller total numbers in cell line systems (11, 40). To our knowledge, these results are also larger than those currently described in any given cell line system in the literature, in any case when considering the amount of protein used and the total measuring time. Furthermore, coverage was extremely consistent between singlet measurements of different cell lines, due to the fact that cell lines tend to have qualitatively similar proteomes (11, 41) and because the depth of proteome coverage reached by our workflow makes our results very robust against 'missing values' that can occur in shotgun proteomics. Although we used a “match between run” strategy in the experiments described here, which resulted in substantial gains, the identification numbers without matching are also very high. Indeed, because of the near absence of sample loss, the maximum amount of peptide material is available for fragmentation and identification. The increased measuring time due to fractionation implies more sequencing events, and thus the nano-fractionator is arguably less reliant on the transfer of peptide identifications.

Various developments can be envisioned to further improve on the results shown here. For instance, the depth of the matching library could be increased, which could be used to reduce the number of fractions without compromising coverage. Although not shown here, the spider fractionator would work equally well with peptide samples that have been derivatized with isotopically labeled mass tags such as iTRAQ or TMT. In this case, a 10-fold decrease in initial sample amount, for instance, would directly translate into a 10-fold reduction in reagent costs. Furthermore, the first dimension column could be further scaled down to enable even smaller sample amounts to be efficiently fractionated and ultra-narrow bore columns and/or ultralow flow rates could also be used in the on-line dimension. Apart from total proteome measurements, the spider fractionator could also be applied to the analysis of post-translational modifications, an area where sensitivity is especially desired. Finally, the scheme presented here is agnostic in regards to acquisition strategies (data-dependent acquisition, data-independent acquisition, or targeted acquisition).

## DATA AVAILABILITY

The RAW and processed data associated with this project is deposited in PRIDE proteomeXchange (project accession number PXD005141).

## Supplementary Material

Supplemental Data

## Footnotes

^1^ The abbreviations used are:

ddH_2_O: double distilled H_2_O
SMC: smooth muscle cell
EC: embryonic carcinoma.
