# Supplementary material for: Loss-less Nano-fractionator for High Sensitivity, High Coverage Proteomics
Source: Mol Cell Proteomics. 2017 Jan 26;16(4):694–705. doi: 10.1074/mcp.O116.065136 (PMC5383787; doi:10.1074/mcp.O116.065136)
Supplement: Supplemental Data [file supp_16_4_694__index.html]

Loss-less nano-fractionator for high sensitivity, high coverage proteomics — Loss-less Nano-fractionator for High Sensitivity, High Coverage Proteomics — Loss-less Nano-fractionator — Supplemental Data 

# Loss-less Nano-fractionator for High Sensitivity, High Coverage Proteomics

## Supplemental Data

- Suppl Figures (.docx, 2.4 MB) - Suppl Figures
- Suppl Table 2 (.xlsx, 15 KB) - Suppl Table 2
- Suppl Table 3 (.xlsx, 13 KB) - Suppl Table 3
- Suppl Table 1 (.xlsx, 15 KB) - Suppl Table 1
