# Supplementary material for: Loss-less Nano-fractionator for High Sensitivity, High Coverage Proteomics
Source: Mol Cell Proteomics. 2017 Jan 26;16(4):694–705. doi: 10.1074/mcp.O116.065136 (PMC5383787; doi:10.1074/mcp.O116.065136)
Supplement: Supplemental Data [file 10.1074_O116.065136_mcp.O116.065136-1.docx]

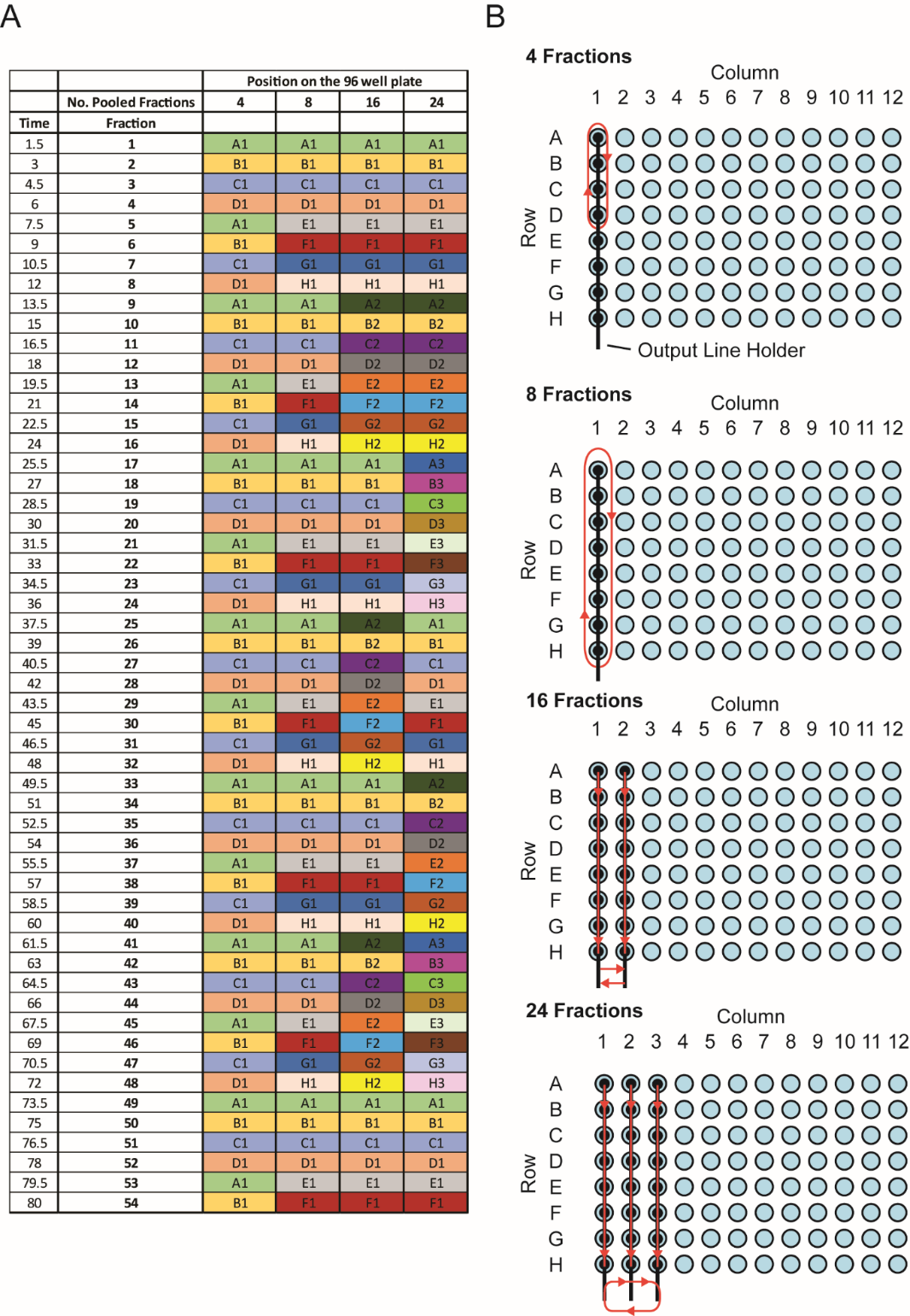


**Supplementary Figure 1: Detailed fractionation and concatenation scheme**. *A,* A 96 well plate annotation scheme is used illustrating sample collection movements. The eight output lines as well as the rotor valve positions (each valve position is always connected to the same output line) have the 96 well plate **row** annotation from **A-H** and the 96 well plate **column** annotation **1-12** is used for the x-axis movement for fractionation into more than 8 fractions. *B*, The collection order of eluting peptides is illustrated for 4, 8, 16 and 24 fractions. If 4 or 8 fractions are desired the output line holder will stay in column 1. Eight shifts of the rotor valve will deposit peptides from the column into each tube of the first column (**A-H**) and the next shift will enter the output line and therefore the first row (**A**) again.


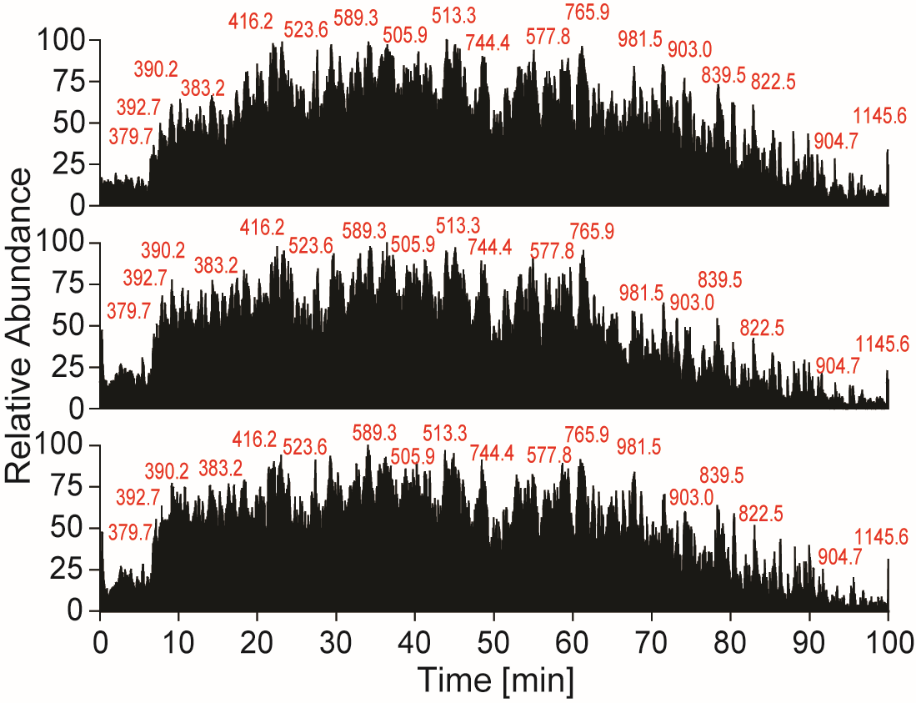


**Supplementary Figure 2: Repeated collection of the same pooled fractions in triplicates with indicated m/z ratios of the same peaks**.


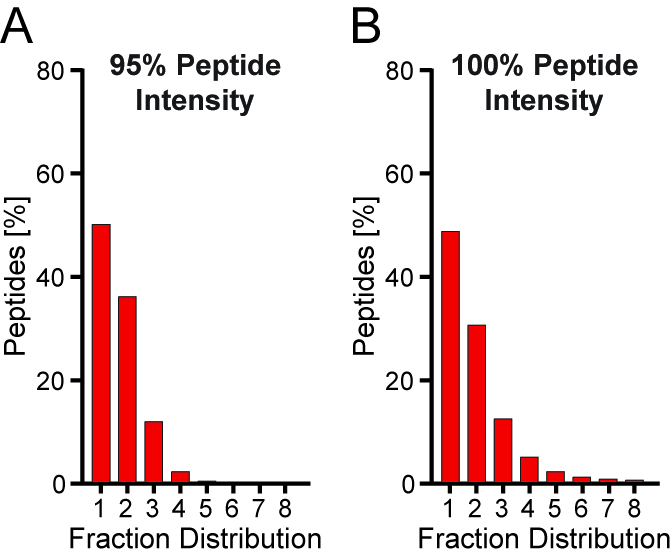


**Supplementary Fig. 3: Fractionation efficiency.** *A*, Histogram of peptides containing at least 95% of their total mass over all fractions in the indicated number of fractions. *B*, Percentage of peptides present in the indicated number of fractions.


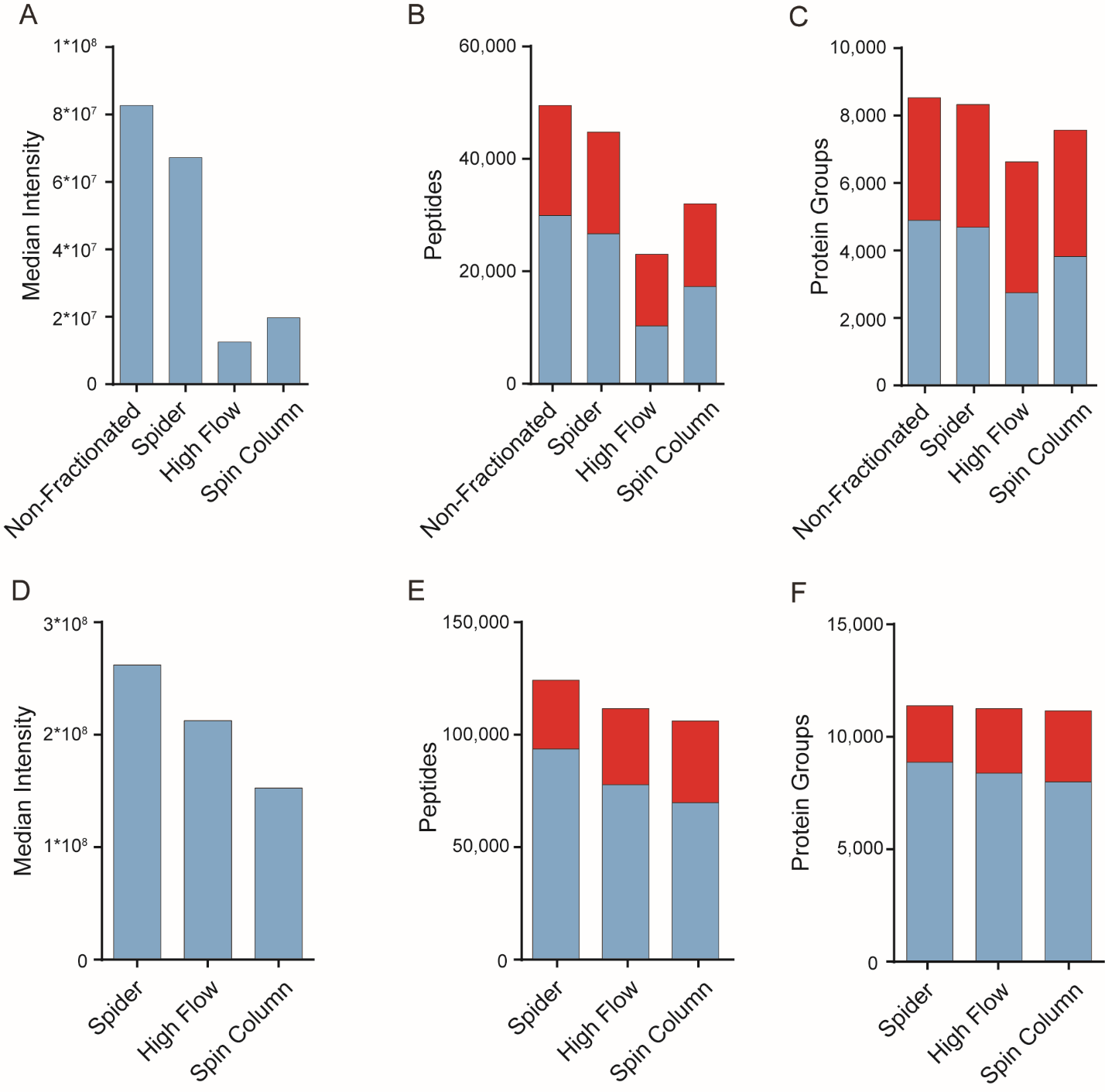


**Supplementary Fig. 4: Comparison of the spider fractionator to other high pH fractionation systems.** *A*, Median peptide intensity of 1 µg fractionated and pooled HeLa peptides for all three fractionation systems and a non-fractionated sample. *B*, Numbers of identified peptides without (blue) and with match between runs (red) for the non-fractionated sample and all three fractionation systems for 1 µg starting material. *C*, Numbers of identified protein groups without (blue) and with match between runs (red) for the non-fractionated sample and all three fractionation systems for 1 µg starting material. *D*, Median peptide intensity of the three fractionation systems for 20 µg starting material. *E*, Numbers of identified peptides without (blue) and with match between runs (red) for all three fractionation systems for 20 µg starting material. *F*, Numbers of identified protein groups without (blue) and with match between runs (red) for all three fractionation systems for 20 µg starting material.
